# Nuclear Markers Reveal Predominantly North to South Gene Flow in *Ixodes scapularis*, the Tick Vector of the Lyme Disease Spirochete

**DOI:** 10.1371/journal.pone.0139630

**Published:** 2015-11-04

**Authors:** Janice Van Zee, Joseph F. Piesman, Andrias Hojgaard, William Cormack Black IV

**Affiliations:** 1 Centers for Disease Control and Prevention, Division of Vector-Borne Diseases, 3150 Rampart Road, Fort Collins, CO, 80521, United States of America; 2 Department of Microbiology, Immunology and Pathology, Colorado State University, Fort Collins, CO, 80523, United States of America; University of Kentucky College of Medicine, UNITED STATES

## Abstract

*Ixodes scapularis*, the tick vector of the Lyme disease spirochete, is distributed over most of the eastern United States, but >80% of all Lyme disease cases occur in the northeast. The role that genetic differences between northern and southern tick populations play in explaining this disparate distribution of Lyme disease cases is unclear. The present study was conducted with 1,155 SNP markers in eight nuclear genes; the 16S mitochondrial gene was examined for comparison with earlier studies. We examined 350 *I*. *scapularis* from 7 states covering a representative area of the species. A demographic analysis using Bayesian Extended Skyline Analysis suggested that *I*. *scapularis* populations in Mississippi and Georgia began expanding 500,000 years ago, those in Florida and North Carolina 200,000 years ago and those from Maryland and New Jersey only during the past 50,000 years with an accompanying bottleneck. Wisconsin populations only began expanding in the last 20,000 years. Analysis of current migration patterns suggests large amounts of gene flow in northern collections and equally high rates of gene flow among southern collections. In contrast there is restricted and unidirectional gene flow between northern and southern collections, mostly occurring from northern into southern populations. Northern populations are characterized by nymphs that quest above the leaf litter, are easy to collect by flagging, frequently feed on mammals such as rodents and shrews, commonly attach to people, and about 25% of which are infected with *B*. *burgdorferi*. If there is a genetic basis for these behaviors, then the patterns detected in this study are of concern because they suggest that northern *I*. *scapularis* populations with a greater ability to vector *B*. *burgdorferi* to humans are expanding south.

## Introduction

The black-legged tick, *Ixodes scapularis* is the main vector of *Borrelia burgdorferi*, the bacterium that causes Lyme disease, the most prevalent vector borne disease in the United States. There are more than 30,000 new cases reported yearly, and possibly ten times that number of cases actually occur [1]. The black-legged tick also transmits the pathogenic agents that cause human granulocytic anaplasmosis [2], Powasson encephalitis [3] and human babesiosis [4]. *Ixodes scapularis* ticks have a wide distribution throughout eastern North America, with populations found along the Atlantic seaboard from Florida to Nova Scotia, and from the Atlantic coast west to the 100^th^ meridian [5]. But risk of Lyme disease spirochete transmission is not uniform throughout this range. Lyme disease cases are focused in the northeastern U.S. (from Maryland to Maine) and in the Midwest (Minnesota-Wisconsin), but are rare or absent in the southeastern U.S. [6, 7]. Interestingly, the behavior of the nymphal stage of *I*. *scapularis* differs dramatically between the northern and southern populations [8]. In the northern U.S., nymphal *I*. *scapularis* quest above the leaf litter, are easy to collect by flagging, frequently feed on mammals such as rodents, commonly attach to people, and about 25% of questing nymphs are infected with *B*. *burgdorferi*. In the south, nymphal *I*. *scapularis* are difficult to collect by flagging, rarely attach to rodents or people, appear to be more common on reptiles than mammals, and *B*. *burgdorferi* infection is extremely rare in questing ticks [9]. Correspondingly, Lyme disease is much more common in the northern U.S. than in the southern U.S. [10].

These and additional morphological differences were considered sufficient to classify northern and southern ticks as distinct species, *I*. *dammini* and *I*. *scapularis*, respectively [11]. However later studies failed to observe any reproductive barrier or chromosomal variation and *I*. *dammini* was reduced to a junior synonym of *I*. *scapularis* [12]. Discrete genetic differences were observed at the protein and DNA level, but were not sufficient to discriminate northern from southern ticks [11–13]. Later studies analyzed the mitochondrial 16S rRNA gene [12–17] and revealed the presence of two distinct clades, one that extends throughout the continental U.S., known as “Clade A” [16] or “All American Clade” [14] and another clade found exclusively in the southern regions, known as “Clade B”[16] or “Southern Clade.”[14] In addition much greater 16S diversity was consistently observed among southern ticks [12–17].

The migration pattern of *I*. *scapularis* ticks has been investigated in several other studies. Some of them focus in specific areas, such as those in Virginia [18] and New York [19], while others have chosen to look at specific genes, especially the 16S mitochondrial gene [20, 21] for signature migration patterns. In a review of the last 30 years of tick population genetic studies [22], the breeding structure of *I*. *scapularis* ticks is described as highly structured on highly mobile hosts [12, 14, 16, 21, 23, 24]. Recently we amplified and sequenced nuclear genes in *I*. *scapularis* using sequence information from the genome project (https://www.vectorbase.org/organisms/ixodes-scapularis) for primer design [17] and to assess the density of Single Nucleotide Polymorphisms (SNPs). We sampled 10 ticks from each of 4 collections from New Jersey, Virginia, Georgia, and Mississippi and analyzed the sequences of 9 nuclear genes and the mitochondrial 16S gene. SNPs were found to be extremely abundant (1 SNP /14 bases). A very preliminary population genetic analysis based on frequencies of 372 SNPs in these 9 genes showed that the ticks fell into three genetic groups. Northern collections from New Jersey and Virginia formed a homogeneous group with low genetic diversity, whereas ticks collected from Georgia and Mississippi formed two groups, each with high genetic diversity. It was also noted that northern ticks appeared to be migrating south but there was little evidence for gene flow in the reverse direction. More recently, the mitochondrial cytochrome oxidase I (COI) and 16S genes and three nuclear genes (serpin2, ixoderin B and lysozyme) were sequenced from field collected northern and southern *I*. *scapularis* [25]. This study also detected a divergence in the mitochondrial gene sequences from some southern specimens. Phylogenetic analyses and analysis of molecular variance (AMOVA) [26] supported significant differences between northern vs. southern populations.

We were intrigued by the patterns indicated by these nuclear marker studies [17, 25] because if there is a genetic basis for the nymphal behaviors that differ between northern and southern populations then northern *I*. *scapularis* populations with a greater ability to vector *B*. *burgdorferi* to humans appear to be expanding south. To assess this possibility, we herein describe a study with sample sizes increased to fifty ticks/collection and the numbers of states sampled increased to seven to cover a more representative area of the distribution of *I*. *scapularis*. Larger samples sizes allowed us to perform a demographic analysis using Bayesian Extended Skyline plots [27] to assess the evolutionary history of *I*. *scapularis* in the U.S and enabled us to perform a non-equilibrium analysis of gene flow [28]. Three genetic groups were recovered when all nuclear genes were analyzed together. Analysis of migration rates and patterns revealed a pattern of very limited gene flow between northern and southern populations with the majority of migration occurring from northern populations into the south.

## Materials and Methods

Collection sites were selected in an opportunistic fashion throughout the natural range of *I*. *scapularis*. Ticks from New Jersey, Wisconsin and North Carolina were collected in 2009 by dragging sheets of cloth across vegetation (“flagging”). Flagging was also used in 2011 to collect from Maryland and in 2012 from Mississippi and Florida. Ticks in the 2012 Georgia collection were taken off of dead deer at a hunter check station. A minimum of 50 ticks were analyzed in each of the seven collections. For Virginia, New Jersey and Mississippi only 40 ticks were collected, since 10 were previously used in another study [17]. No specific permissions were required for these locations/activities, all ticks were collected by collaborators on public property and did not involve the use of live vertebrate hosts.

**Fig 1:**
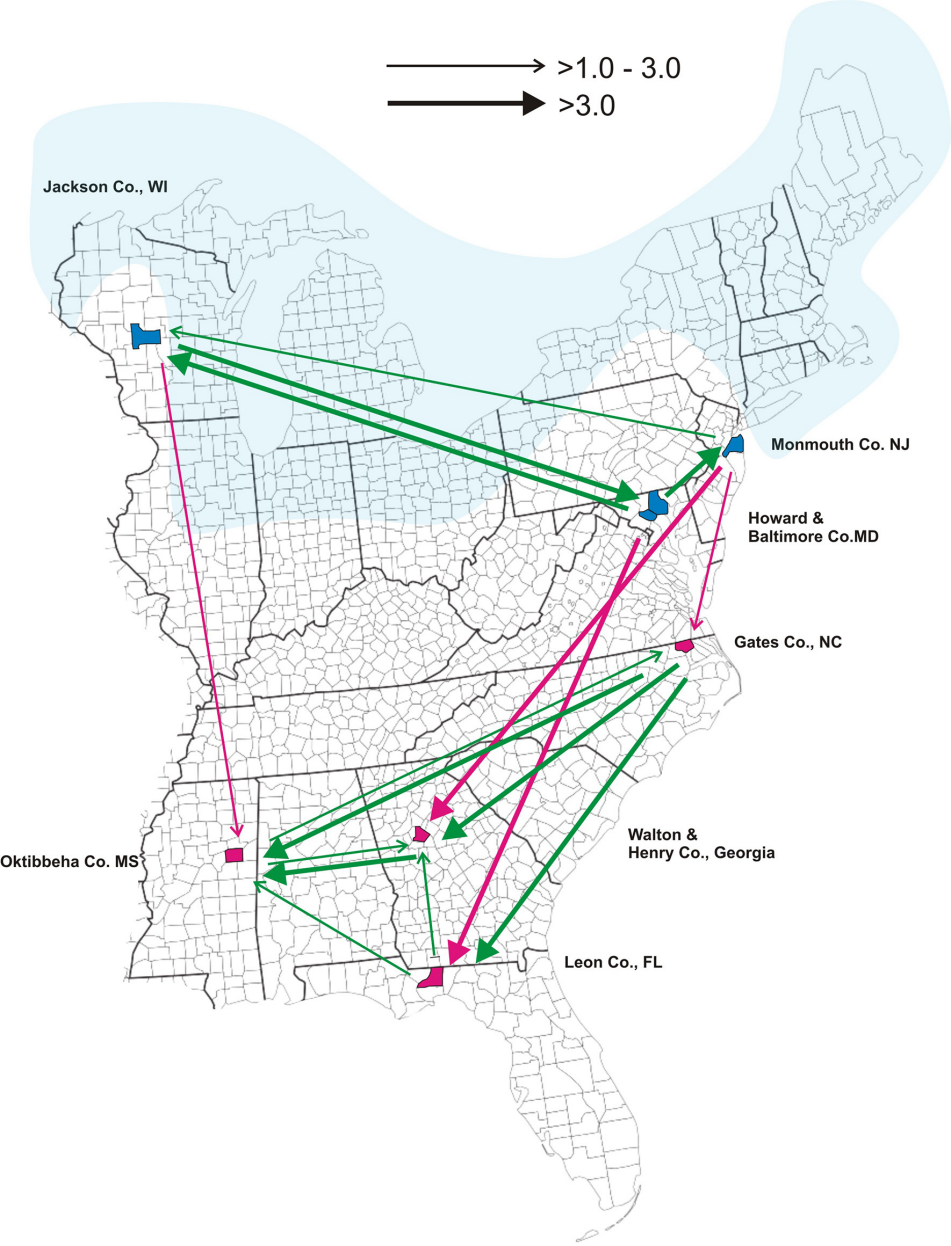
Locations of tick collections from Monmouth County, New Jersey, Jackson County, Wisconsin, Gates County, North Carolina, Howard and Baltimore Counties, Maryland, Oktibbeha County, Mississippi, Leon County, Florida and from Walton and Henry Counties, Georgia. The Laurentide Ice Sheet existed from 95,000 and 20,000 years before the present day. Its maximum intrusion is highlighted in blue. The arrows in the diagram indicate the rate and direction of gene flow among the seven locations as estimated by LAMARC. *4N*_*e*_ *m* values are displayed as arrows on the collecting site map (Fig 1) to indicate the direction of gene flow between two collections and relative rates of immigration. When *4N*_*e*_ *m* ≤ 1, no arrow was displayed, but when 1 < *4N*_*e*_ *m* ≤ 3.0 a thin open arrow is displayed. Thick open arrows indicate when *4N*_*e*_ *m* ≥ 3. Green arrows signify gene flow within the south or within the north while red arrows indicate north to south gene flow.

The counties where collections occurred are mapped in Fig 1. For this study and in keeping with all previous population genetic studies of this species [12–17] New Jersey, Maryland and Wisconsin are considered northern while North Carolina, Georgia, Mississippi and Florida are southern.

Nine genes were used for this study: Ixolaris 2A, Defensin, Heat shock protein 70 b2, Trospa, Ferredoxin-glutamate synthase, Dihydropyrimidine dehydrogenase, Serotonin 4 receptor, Acetylcholinesterase and 16S. Extensive details on gene selection, DNA isolation, primer design, PCR conditions, and sequencing protocols are published [17]. Sequences were aligned using CLUSTALW (www.genome.jp/tools/clustalw) and visually inspected to insure correct alignment along codons. The phase unknown sequences of each of the 9 genes were entered in “fas” format into DnaSP v5 [29], where each was converted with the PHASE program [30] into a pair of phase-estimated sequences. DnaSP v5 was used to calculate S, the number of SNPs appearing in more than one sequence, η, the overall number of SNPs (S including singletons) and H, the numbers of unique alleles. π is the average observed number of pairwise differences per nucleotide between all pairs of sequences. θ, calculated from η, is the average expected number of pairwise differences per nucleotide between all pairs of sequences. θ also estimates the approximate product of effective population size (*N*_*e*_) and the neutral mutation rate per site per generation (μ) and therefore estimates the approximate maximum expected amount of genetic variation in a natural population. Tajima’s D [31] is the normalized difference between π and θ. A negative Tajima’s D indicates fewer pairwise differences between sequences than expected and can arise from removal of variation through purifying selection or through a recent population expansion. A positive Tajima’s D indicates more pairwise differences between sequences than expected and can arise from balancing selection or through a recent population contraction.

All aligned haplotypes were compared using RAxML v. 8 [32] to identify identical haplotypes and determine haplotype frequencies for mismatch analysis [33] and AMOVA [26] analyses by ARLEQUIN version 3.01 [34]. A FORTRAN program was written to extract SNPs from each gene and place these in a format for analysis by STRUCTURE 2.3.4 [35] which was then used to estimate the numbers of genetic clusters (K) of ticks on the basis of their genotypes at the 1,155 SNPs. Population identity was not used as a prior. The log probability of the data (X) was estimated for each value of K (conditional probability = (X|K)). For K = 1–14, the burnin period was 100,000 while the number of MCMC replicates after burnin was 900,000 [36]. Each run was repeated 10 times for each value of K for all 8 nuclear genes combined. Repeating the analysis with 20 repetitions for K = 1–5 did not change the estimate of K. The ΔK method [37] estimated the most likely value of K. STRUCTURE also quantifies the likelihood that each tick belongs to each of the K clusters and assigns ticks to a cluster based upon this information. The program DISTRUCT [38] was used to display membership likelihood coefficients. Cluster membership was represented as different colors, and individual ticks were depicted as vertical bars partitioned into segments that correspond to membership coefficients in each of the seven collection sites.

Maximum clade credibility trees were estimated for the 8 nuclear genes combined and for the 16S using the Bayesian Evolutionary Analysis Sampling Trees (BEAST v1.8.1) package [27] and by MrBayes 3.2.3 [39] (http://mrbayes.sourceforge.net/download.php). Results were visually compared to assess whether MrBayes and BEAST produce similar trees. The Bayesian Evolutionary Analysis Utility (BEAUti v1.8.1) in BEAST [27] generated ten million coalescent trees based upon the Kingman Coalescent Constant Size model [40]. The (HKY) substitution model [41] was used with empirical nucleotide frequencies. A maximum clade credibility tree was then derived from these many trees using TreeAnnotator (v1.8.1) in http://beast.bio.ed.ac.uk/treeannotator in BEAST with 3% of trees used during burnin. FigTree v1.4.2 in BEAST was used to view the maximum clade credibility tree and to display the posterior probability (PP) for each branch.

Extended Bayesian Skyline Plot (EBSP) analysis [27] was also performed with BEAST. EBSP is a relatively new variable-dimension Bayesian phylogenetic method for inferring non-parametric population size changes over geological time from multiple loci [42]. The EBSP estimates *N*_*e*_ changes through time by directly inferring *N*_*e*_ as a function of time using sequence data. We used EBSP to assess the evolutionary history of *I*. *scapularis*.

The 8 nuclear gene sequences were next entered as a group for analysis of *θ* and the effective migration rate *4N*_*e*_ *m* among collections using LAMARC 2.1.10 [28, 43]. In LAMARC, the immigration parameter *M* equals *m* (the immigration rate per generation) divided by μ. If *M* = 1, the rate that new alleles enter a population through immigration is equal to the rate new alleles arise by mutation. The effective migration rate *4N*_*e*_ *m* was calculated between a donor and recipient population as *M* multiplied by the recipient population *θ*. A *4N*_*e*_ *m* < 1 suggests that immigration is insufficient to offset the effects of genetic drift so that populations diverge in allele frequencies while a *4N*_*e*_ *m* > 1 suggests that there is sufficient immigration to offset the effects of genetic drift to maintain homogeneity among populations. *4N*_*e*_ *m* values are displayed as arrows on the collecting site map (Fig 1) to indicate the direction and rate of gene flow between two collections. When *4N*_*e*_ *m* ≤ 1, no arrow was displayed, but when 1 < *4N*_*e*_ *m* ≤ 3.0 a thin open arrow is displayed. Thick open arrows indicate when *4N*_*e*_ *m* ≥ 3. Green arrows signify gene flow within the south or within the north while red arrows indicate north to south gene flow (south to north gene flow was never detected).

## Results

### Genetic diversity

**Fig 2:**
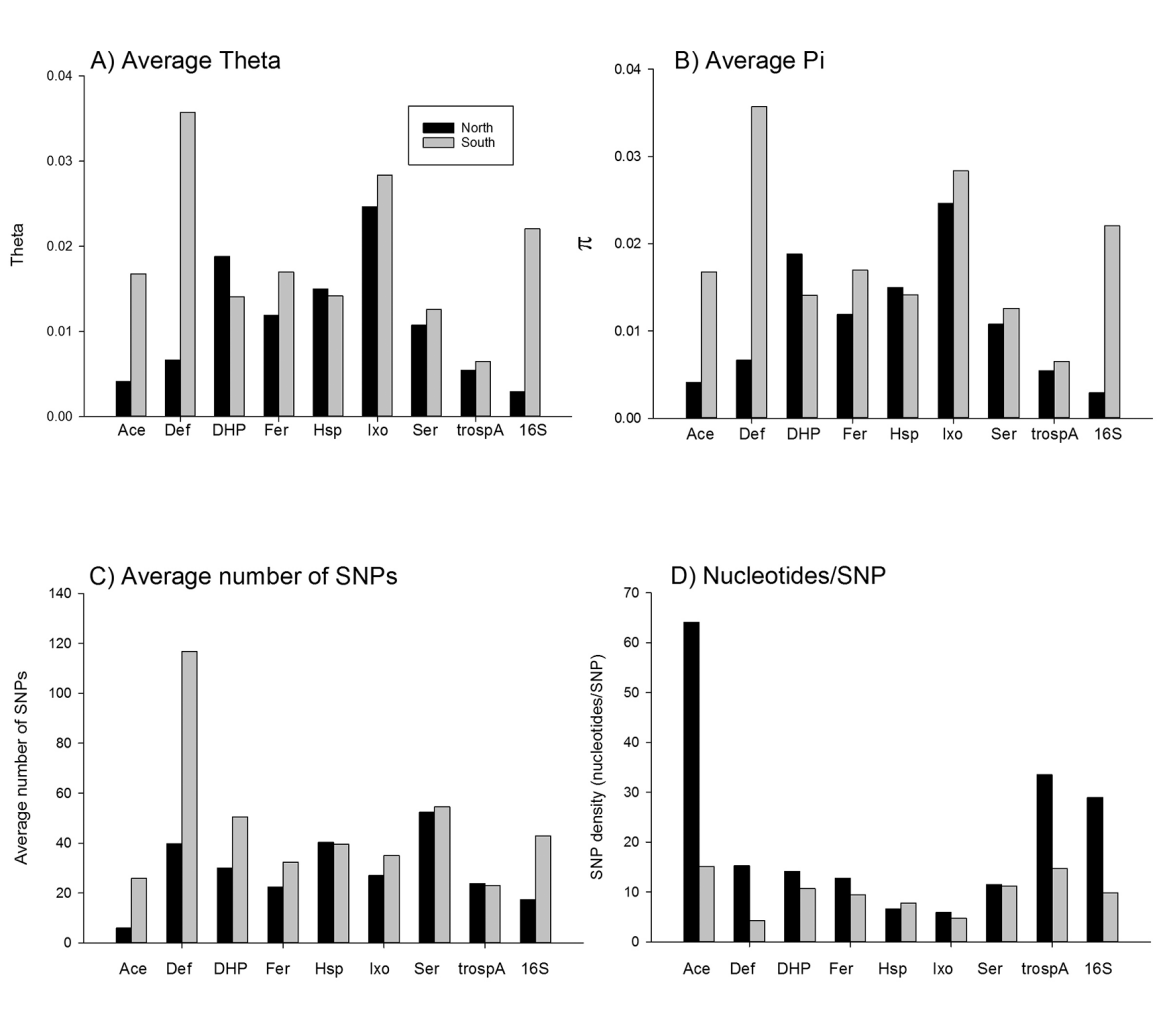
Measurement of genetic diversity among the 8 nuclear markers and at the 16S gene. A), average θ values and B) π values, C) The average numbers of SNPs and D) SNP density/ nucleotides.

Table 1 provides the full name of each of the eight nuclear genes and their VectorBase accession number. Also listed are the region(s) of the gene examined, the length of the regions, the VectorBase coordinates for the beginning and end of each region and the GenBank accession numbers. The number of nucleotides in coding versus intronic regions is listed as well. Table 2 lists the number of sequences obtained for each gene in the overall study and in each of the seven collections. S is the number of SNPs appearing in more than one sequence and η is the overall number of SNPs (including singletons) while H is the number of unique alleles. π is the average observed number of pairwise differences per nucleotide. θ, calculated from η, is the average expected number of pairwise differences per nucleotide. Tajima’s D is the normalized difference between π and θThe significance of D is indicated by *p<0.05, **p<0.01, ***p<0.001. The D ratio is only calculated for exons and is D for replacements substitutions divided by D for silent substitutions. Fig 2 compares measures of genetic diversity among the 8 nuclear markers and at the 16S gene. With the exceptions of *Dhp* and *Hsp*, average θ values and π values were greater in southern collections (Fig 2A and 2B). The average numbers of SNPs detected were greater among southern collections in all genes except *Hsp* and *TROSPA* (Fig 2C). Similarly SNP density was greatest in southern collections in all genes except *Hsp* and *Ser* (Fig 2D). With the exception of *Ace* all of Tajima’s D values in Table 2 are negative implying removal of variation through purifying selection or through a recent population expansion. Purifying selection is unlikely since the absolute value of most (41/54) of Tajima's D (Nonsynonymous/Synonymous) ratios are > 1. Recent population expansion is a more likely explanation and is supported in subsequent analyses.

**Table 1:** Gene name, GenBank and VectorBase accession number for each of the 8 nuclear *Ixodes scapularis* genes and the accession number for the 16S gene. Under each gene name are listed the region of the gene examined, the length of that region, the GenBank accession number and the VectorBase coordinates for the beginning and end of each sequence. The numbers of nucleotides in coding versus intronic regions are listed.

Gene name	VectorBase Accession #	Gene region	Start	End	Coding	Intronic	Total	GenBank Accession
Acetylcholinesterase (ace)	ISCW003278	Exon 1	31	351	320	0	320	KT847953—KT848578
Dihydropyrimidine dehydrogenase (dhp)	ISCW022746	Exon 17	40	375	206	130	336	KT849259—KT849838
TROSPA receptor (trospa)	ISCW011465	Exon 2	79	411	332	0	332	KT850389—KT851034
Serotonin 4 receptor (ser)	ISCW007619	Exon 1	52	643	591	0	591	KT846601—KT847274
Defensin (def)	ISCW022102	Exon 4	29	485	114	342	456	KT849839—KT850388
Heat shock protein 70 b2 (hsp)	ISCW015267	Exon 1	12	273	261	0	261	KT847275—KT847952
Ixolaris 2A (ixo)	ISCW014813	Exon 2	20	176	156	0	156	KT845965—KT846600
Ferredoxin-glutamate synthase (fer)	ISCW005873	Exon 15	19	359	340	0	340	KT848579—KT849258
16S mtDNA (16s)	NC_002010		12,112	12,467	355	0	355	KT851035—KT851374

**Table 2:** Summary of sample sizes and variability in the nine genes in each of the seven collection sites.

Collection	Gene	N	S	η	H	π	θ	Tajima's D	D ratio (Rep./Silent)
Florida	ace	49	11	12	11	0.0043	0.0073	-1.076	0.57
Florida	dhp	44	69	100	48	0.0196	0.0593	-2.23**	1.33
Florida	trosp	50	19	20	20	0.0056	0.0117	-1.521	0.81
Florida	ser	50	65	72	52	0.0143	0.0235	-1.275	2.63
Florida	def	35	73	80	34	0.0288	0.0402	-0.961	29.36
Florida	hsp	50	31	36	42	0.0137	0.0266	-1.502	1.57
Florida	ixo	48	34	36	42	0.0224	0.0449	-1.561	1.17
Florida	fer	49	26	28	38	0.0183	0.0195	-0.189	-1.25
Florida	16s	49	51	54	13	0.0299	0.0351	-0.511	
Georgia	ace	45	38	41	32	0.0253	0.0255	-0.027	-0.35
Georgia	dhp	37	24	25	23	0.0072	0.0194	-1.944*	1.29
Georgia	trosp	42	23	23	25	0.0062	0.0140	-1.687	6.4
Georgia	ser	46	49	52	51	0.0129	0.0171	-0.798	4.69
Georgia	def	33	92	134	40	0.0480	0.0683	-1.032	5.04
Georgia	hsp	44	33	39	52	0.0132	0.0296	-1.758	1.18
Georgia	ixo	45	27	29	25	0.0306	0.0367	-0.509	-7.19
Georgia	fer	50	32	39	30	0.0131	0.0270	-1.601	3.42
Georgia	16s	47	34	39	21	0.0229	0.0252	-0.303	
Mississippi	ace	44	20	21	15	0.0123	0.0130	-0.15	-0.82
Mississippi	dhp	42	31	43	33	0.0116	0.0257	-1.756	1.47
Mississippi	trosp	43	28	28	19	0.0071	0.0171	-1.795*	0.9
Mississippi	ser	46	63	73	45	0.0120	0.0243	-1.66	1.15
Mississippi	def	33	69	75	27	0.0255	0.0379	-1.116	2.99
Mississippi	hsp	50	60	72	59	0.0206	0.0533	-1.993*	1.46
Mississippi	ixo	37	51	58	48	0.0401	0.0763	-1.572	1.19
Mississippi	fer	47	49	59	50	0.0200	0.0413	-1.666	7.45
Mississippi	16s	49	57	59	16	0.0238	0.0387	-1.341	
North Carolina	ace	29	33	35	23	0.0252	0.0237	0.21	-0.85
North Carolina	dhp	48	38	50	41	0.0179	0.0290	-1.221	12.86
North Carolina	trosp	48	22	24	27	0.0072	0.0142	-1.469	0.79
North Carolina	ser	46	41	42	36	0.0114	0.0138	-0.557	0.63
North Carolina	def	44	99	113	51	0.0407	0.0560	-0.912	7.7
North Carolina	hsp	48	23	29	38	0.0093	0.0216	-1.741	1.16
North Carolina	ixo	49	29	35	38	0.0203	0.0435	-1.648	1.4
North Carolina	fer	50	22	28	50	0.0165	0.0194	-0.449	-5.79
North Carolina	16s	50	22	24	14	0.0116	0.0153	-0.799	
Maryland	ace	50	3	3	3	0.0034	0.0018	1.573	n.a.
Maryland	dhp	33	51	53	31	0.0211	0.0331	-1.215	1.66
Maryland	trosp	46	14	14	13	0.0049	0.0084	-1.164	1.4
Maryland	ser	46	44	45	32	0.0105	0.0148	-0.93	2.14
Maryland	def	44	43	43	17	0.0074	0.0201	-2.009*	n.a.
Maryland	hsp	49	34	38	39	0.0139	0.0282	-1.579	1.97
Maryland	ixo	49	31	40	47	0.0226	0.0497	-1.71	0.67
Maryland	fer	50	19	19	27	0.0099	0.0132	-0.713	0.68
Maryland	16s	50	10	10	12	0.0033	0.0064	-1.39	
New Jersey	ace	50	8	9	12	0.0041	0.0054	-0.595	-0.01
New Jersey	dhp	41	43	45	37	0.0171	0.0272	-1.198	1.02
New Jersey	trosp	46	56	63	32	0.0084	0.0374	-2.514***	1.07
New Jersey	ser	49	62	67	33	0.0115	0.0220	-1.552	1.05
New Jersey	def	46	22	23	23	0.0040	0.0102	-1.826*	1.13
New Jersey	hsp	49	46	58	58	0.0185	0.0431	-1.832*	1.91
New Jersey	ixo	41	29	35	39	0.0307	0.0451	-1.009	-2.22
New Jersey	fer	50	27	29	35	0.0125	0.0201	-1.143	3
New Jersey	16s	50	8	8	12	0.0033	.0052	-0.978	
Wisconsin	ace	47	7	7	11	0.0048	0.0043	0.311	-0.66
Wisconsin	dhp	46	54	81	45	0.0183	0.0473	-2.02*	0.87
Wisconsin	trosp	46	7	7	8	0.0031	0.0042	-0.582	1.25
Wisconsin	ser	46	51	65	52	0.0104	0.0214	-1.664	1
Wisconsin	def	40	35	37	31	0.0086	.0169	-1.56	-2.28
Wisconsin	hsp	49	35	47	60	0.0125	0.0349	-2.035*	1.09
Wisconsin	ixo	49	21	23	23	0.0207	0.0286	-0.819	1.5
Wisconsin	fer	44	21	21	30	0.0134	0.0149	-0.309	-3.06
Wisconsin	16s	45	8	8	9	0.0022	0.0052	-1.623	
All	ace	314	61	68	81	0.0134	0.0307	-1.577	0.54
All	dhp	291	110	181	148	0.0166	0.0988	-2.447***	1.11
All	trosp	323	95	113	96	0.0098	0.0493	-2.304**	1.14
All	ser	329	159	201	238	0.0124	0.0480	-2.172**	1.25
All	def	275	162	226	168	0.0298	0.0857	-1.935*	1.57
All	hsp	339	100	136	100	0.0148	0.0734	-2.307**	1.19
All	ixo	318	87	122	205	0.0282	0.1112	-2.155**	1.61
All	fer	340	78	103	202	0.0172	0.0520	-1.911*	2.11
All	16s	340	106	116	72	0.0210	0.0531	-1.813*	

N is number of sequences obtained for each gene in the overall study and in each of the seven collection sites. S is the number of SNPs appearing in more than one sequence and η is the overall number of SNPs (including singletons) while H is the number of unique alleles. π is the average observed number of pairwise differences per nucleotide. θ, calculated from η, is the average expected number of pairwise differences per nucleotide. Tajima’s D is the normalized difference between π and θ. The significance of D is indicated by

*p<0.05

**p<0.01

***p<0.001.

The D ratio is only calculated for exons and is D for replacements substitutions divided by D for silent substitutions.

Table 3 lists the numbers of unique alleles identified among all collections in the 8 nuclear markers and in the 16S gene. Also listed are the numbers of alleles that occurred in common between northern and southern collections and the number that were unique to southern or northern collections. There were from 0.70–3.2 fold more alleles detected among southern as compared to northern collections. Table 3 also shows that, with the exception of *hsp*, only a small percentage of the alleles were shared between northern and southern collections, a pattern consistent with limited gene flow.

**Table 3:** Number of alleles at each gene (H from Table 2) and the number and percentage of alleles shared in common between and among northern (MD, NJ, WI) and southern (FL,MS,GA,NC) collections. The numbers of alleles unique to the north and to the south are listed.

Gene	Number of alleles	Number in common in north & south	% in common	Number unique to south (FL,MS,GA,NC)	Number unique to north (MD,NJ,WI)
*ace*	92	7	7.6%	69	16
*dhp*	89	7	7.9%	42	40
*trospa*	103	5	4.9%	61	37
*ser*	238	20	8.4%	135	83
*def*	189	13	6.9%	123	53
*hsp*	61	27	44.3%	16	18
*ixo*	204	18	8.8%	111	75
*fer*	199	13	6.5%	126	60
Total	1175	110	9.4%	683	382
*16S*	105	7	6.7%	67	31

AMOVA analyses of the 16S sequences (Table 4A) show that 11.8% of the variance arose between northern and southern collections while 34.5% arose among collections in the north and in the south. Most (53.7%) of the variation arose within individual collections. In contrast, AMOVA analyses of individual nuclear genes (Table 4B) showed that on average 15.5% of the variance arose between northern and southern collections but this varied from 0–62% among genes while only 6.0% arose among collections in the north and in the south and this varied from 1–22% among genes. Most (78.5%) of the variation arose within individual collections.

**Table 4:** Analysis of molecular variance comparing variation between and within our northern and southern collections of *Ixodes scapularis*. Significant P values are highlighted in gray.

**A) 16S**				
Source of variation	d.f.	Sum of Squares	F(prob)	%
Between North and South	1	218.0	0.1176	11.8
Among collections w/i North or South	5	509.7	0.3911***	34.5
Within collection	336	1055.5	0.4627***	53.7
Total	342	1783.2		100.0
**B Nuclear genes**	** **	** **	** **	** **
Source of variation	d.f.	Sum of Squares	F(prob)	%
Between North and South				15.5
Ace	1	97.9	0.0679	6.8
Def	1	642.5	0.2170*	21.7
Dhp	1	183.21	0.1628*	16.3
Fer	1	154.336	0.1608*	16.1
Hsp	1	4.51	-0.0016	-0.2
Ixo	1	11.016	-0.0073	-0.7
Ser	1	38.235	0.0192	1.9
TrospA	1	532.928	0.6230*	62.3
Among collections w/I North or South				6.0
Ace	5	232.4	0.2333***	21.8
Def	5	132.4	0.0298**	2.3
Dhp	5	85.248	0.0564***	4.7
Fer	5	83.573	0.0699***	5.9
Hsp	5	27.684	0.0193***	1.9
Ixo	5	76.3	0.0719***	7.3
Ser	5	75.478	0.0339***	3.3
TrospA	5	12.803	0.0167***	0.6
Within collection				78.5
Ace	621	1031.6	0.2854***	71.5
Def	554	4270.9	0.2403***	76.0
Dhp	566	1642.411	0.2101***	79.0
Fer	665	1353.094	0.2195***	78.1
Hsp	667	1278.45	0.0177***	98.2
Ixo	610	1196.555	0.0652***	93.5
Ser	664	2297.306	0.0525***	94.8
TrospA	635	636.241	0.6293***	37.1
Total				
Ace	627	1362.0		100.0
Def	560	5045.8		100.0
Dhp	572	1910.869		100.0
Fer	671	1591.003		100.0
Hsp	673	1310.644		100.0
Ixo	616	1283.872		100.0
Ser	670	2411.019		100.0
TrospA	641	1181.972		100.0

*P<0.05.

**P<0.001,

***P<0.0001

**Fig 3:**
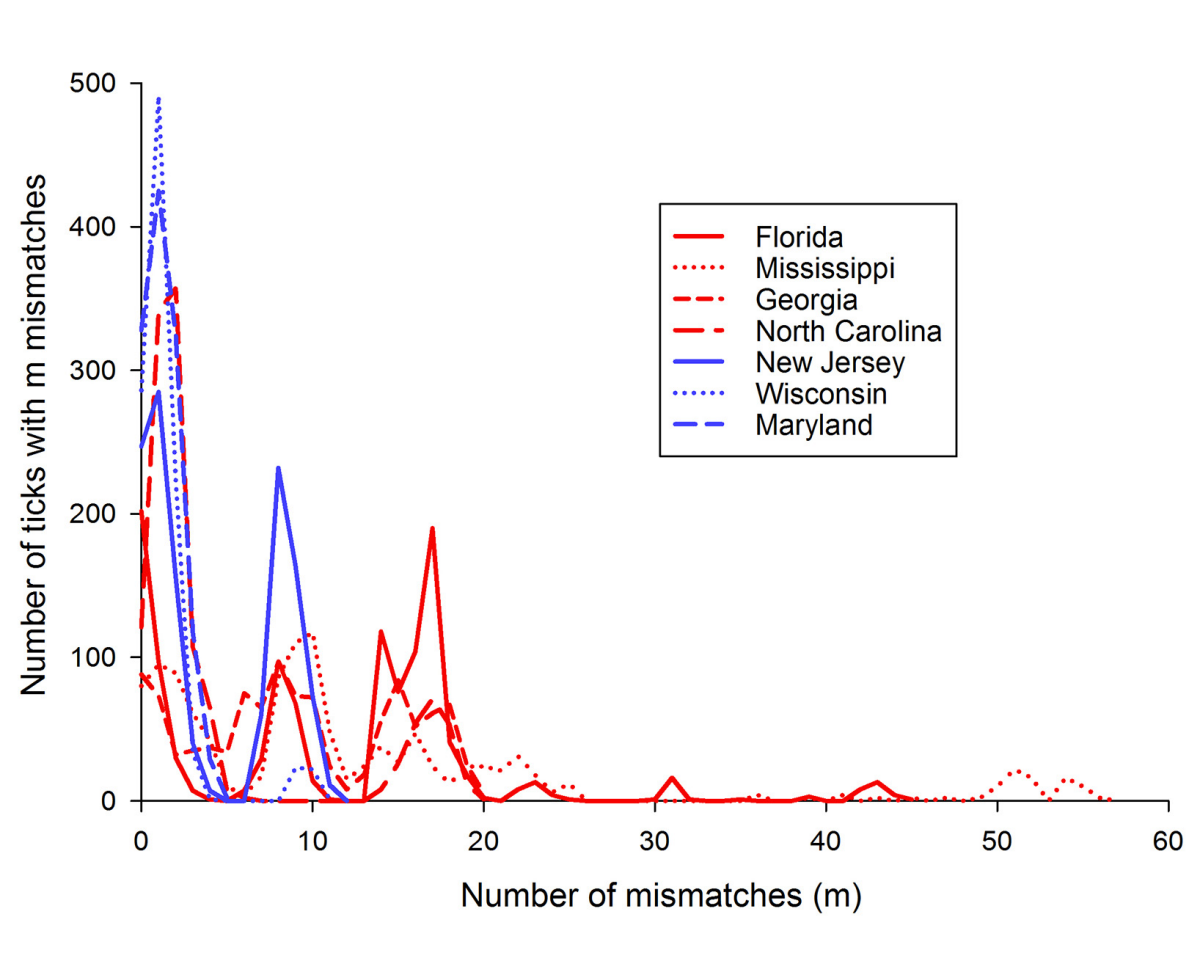
Mismatch distributions among the four southern collections (in red) and the three northern collections in blue.

A mismatch distribution is the frequency distribution of the observed number of differences between pairs of haplotypes [33]. A unimodal distribution with a small range in numbers of mismatches is indicative of populations that have gone through a recent demographic expansion. In contrast, a multimodal distribution involving a large range in the number of mismatches is indicative of a population at demographic equilibrium reflecting highly stochastic shape of gene trees [33]. Fig 3 shows that mismatch distributions among the four southern collections (in red) are multimodal with 0–57 mismatches while the northern collections (in blue) have no more than 11 mismatches and are unimodal with the exception of New Jersey which has two large peaks but without a large range of mismatches possibly suggesting two expansions. Thus southern *I*. *scapularis* populations appear to be at a demographic equilibrium while northern populations have gone through one or in the case of New Jersey two recent demographic expansions. Demographic expansion was also supported by analysis of Tajima’s D (Table 2).

### Phylogenetic relationships among *I*. *scapularis* collections

**Fig 4:**
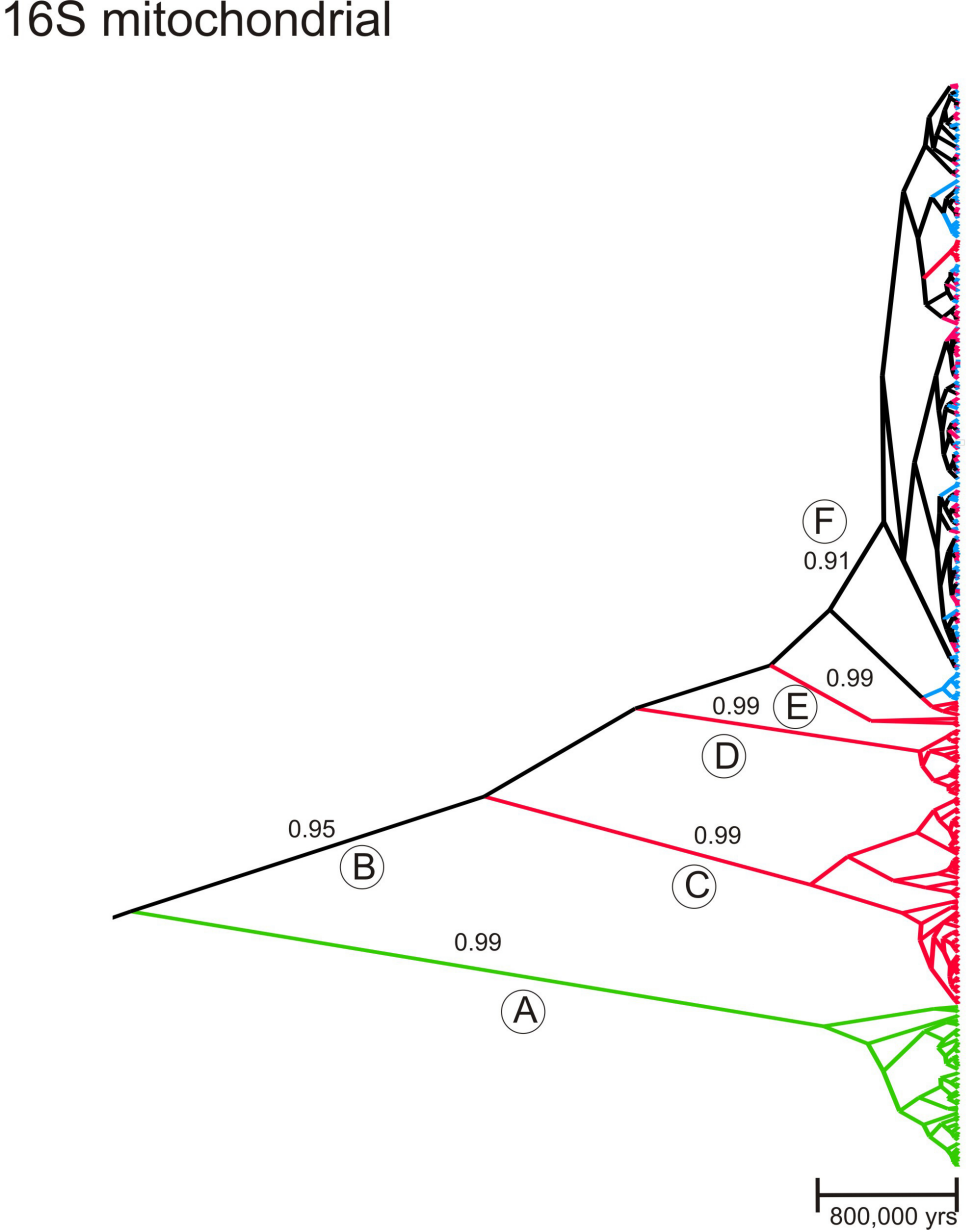
Maximum clade credibility coalescent tree among the mitochondrial 16S sequences using *Ixodes ricinus* as an outgroup. The time scale is based upon a clock rate for the 16S arthropod gene of 1.06% /million years (My). Numbers above some branches are posterior probabilities (PP) for values ≥ 0.90. Sequences collected exclusively in the south are colored red while those only collected in the north appear in blue. Those found in both the north and south appear in black and *I*. *ricinus* sequences appear in green. Any high order branches that join both north and south sequences appear in black. Some branches are labelled with a capital letter in a circle to assist discussion.

Phylogenetic relationships among all of the 16S sequences generated in the present study and using *Ixodes ricinus* as an outgroup were assessed using BEAST. A clock rate for the 16S arthropod gene was set at 1.06% /million years (My) [44]. The maximum clade credibility coalescent tree appears in Fig 4. Only clades with a posterior probability (PP) ≥ 0.90 are indicated. *Ixodes ricinus* sequences appear in green (clade **A**). Sequences collected exclusively in the south are colored red (e.g. clade **C-**E) while those only collected in the north are blue. Any high order branches that join both north and south sequences are black (clades B and F). The *I*. *scapularis* clade B has a PP = 0.95, the next three clades C-E are exclusively southern (red) in composition with PP = 0.99. Clade F (PP = 0.91) contains the remainder of all sequences and is a mixture of ticks collected in both the north and south. Note that this tree implies historical, but not necessarily current, unidirectional introgression of southern ticks into the north since no blue sequences appear in southern clades C-E. The topology of the maximum clade credibility tree and PP are extremely similar to those generated by MrBayes (tree not shown).

**Fig 5:**
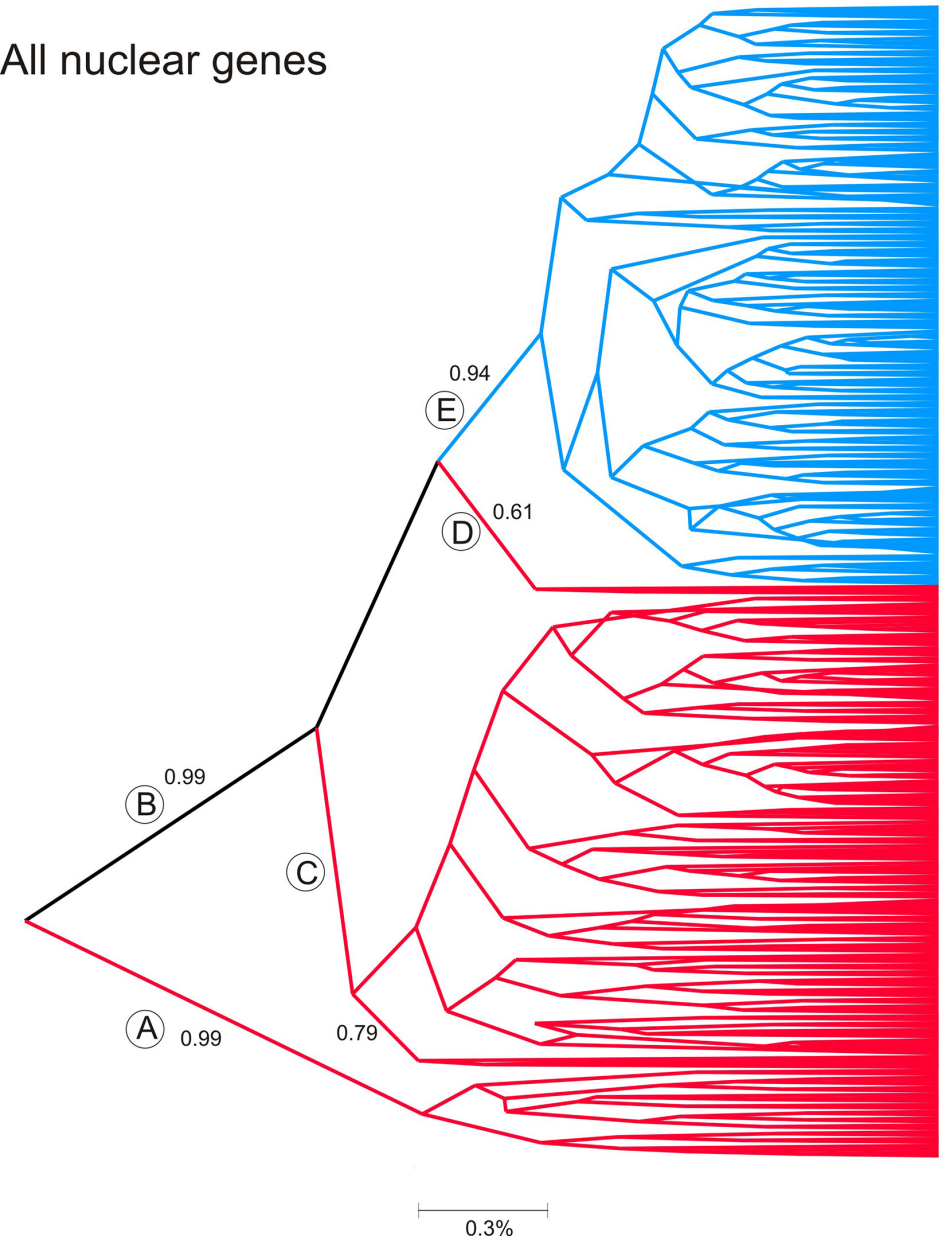
Maximum clade credibility coalescent tree derived all combined genes in the 338 ticks for which we obtained complete sequences in all genes. Numbers above some branches are posterior probabilities (PP) for values ≥ 0.90. Sequences collected exclusively in the south are colored red while those only collected in the north appear in blue. Those found in both the north and south appear in black. Any high order branches that join both north and south sequences appear in black. Some branches are labelled with a capital letter in a circle to assist discussion.

Maximum clade credibility coalescent trees were next derived for the combined nuclear genes but without outgroups since these genes have not been sequenced in other acari (Fig 5). This tree, as with the 16S tree, also has three southern branches A, C, D. Clade A has a PP of 0.99, while C and D arise from clade B (PP = 0.99) that contains the remainder of the sequences. Most southern ticks arose in clade C (PP = 0.79) while only two southern ticks arose at the base (clade D) of the blue clade E (PP = 0.94). Northern ticks were monophyletic (PP = 0.94). This tree implies little to no current gene flow between northern and southern ticks.

**Fig 6:**
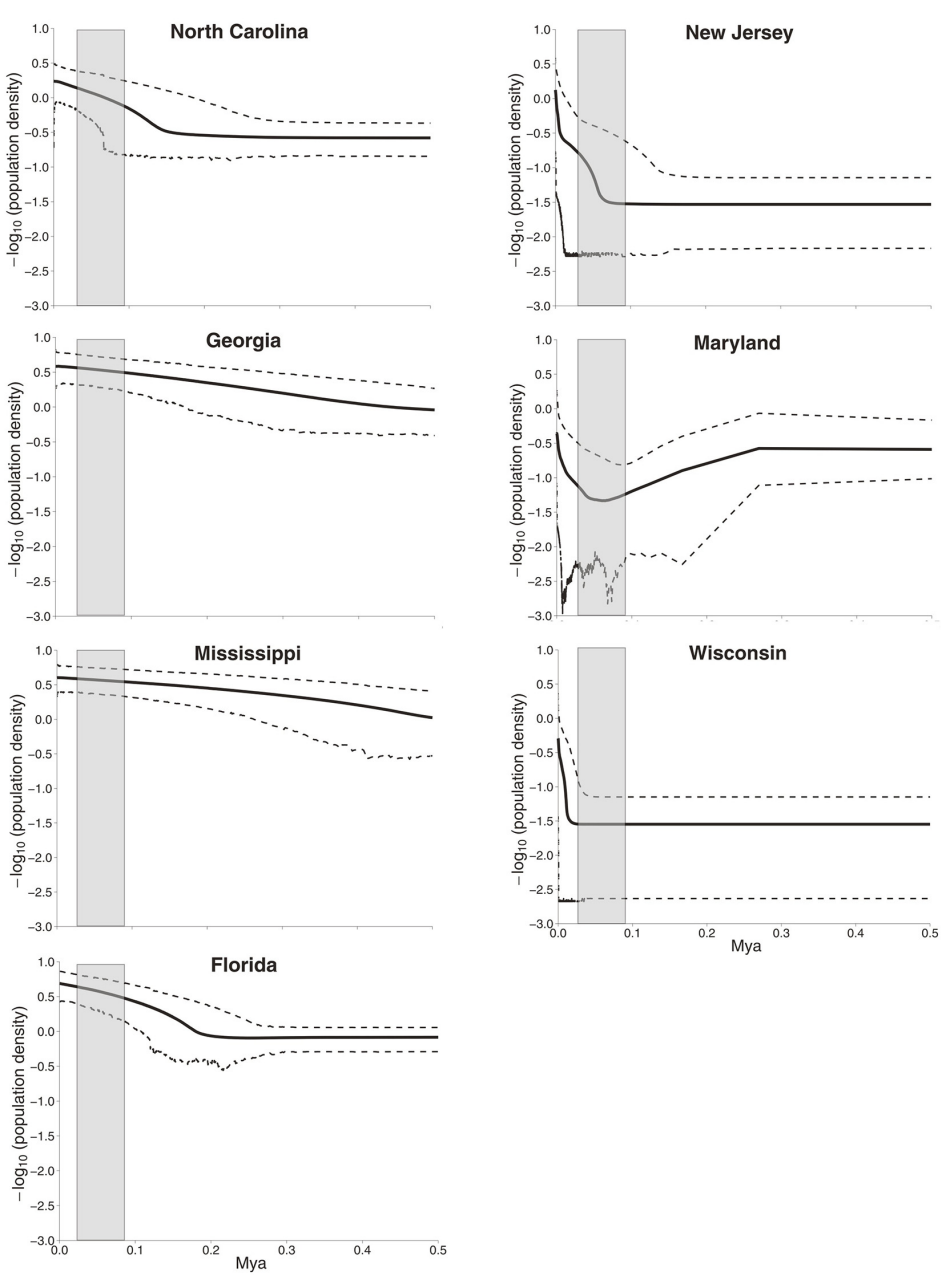
Extended Bayesian Skyline Plot (EBSP) analysis of population size changes over geological time estimated from all 9 loci. An EBSP was derived for each of the 7 collections which were then plotted on a single graph.

Extended Bayesian Skyline Plot (EBSP) [42] was performed with all nuclear genes combined following the online tutorial provided by J. Heled (http://beast-mcmc.googlecode. com/svn-history/r3615/trunk/doc/EBSP). The authors demonstrate the essential role of multiple loci in recovering population size dynamics. Multi-locus data recover past bottlenecks that cannot be characterized by analysis of a single locus.[42] EBSP are read from right to left for each of the collections and the period during which the Laurentide Ice Sheet existed is indicated in gray (Fig 6). *Ixodes scapularis* populations in Mississippi and Georgia began expanding 500,000 years ago, those in Florida and North Carolina 200,000 years ago and those from Maryland and New Jersey only during the past 50,000 years with an accompanying bottleneck. Wisconsin populations only began expanding in the last 20,000 years. This demographic analysis is consistent with a hypothesis that *I*. *scapularis* arose as a species in the southeastern U.S., expanded further south to Florida and North to the Carolinas and only within the last 50,000 years became established in the Northeast. Northern Midwest and Canadian populations only became established as the Laurentide Ice Sheet retreated.

### Genetic analysis of individual ticks

**Fig 7:**
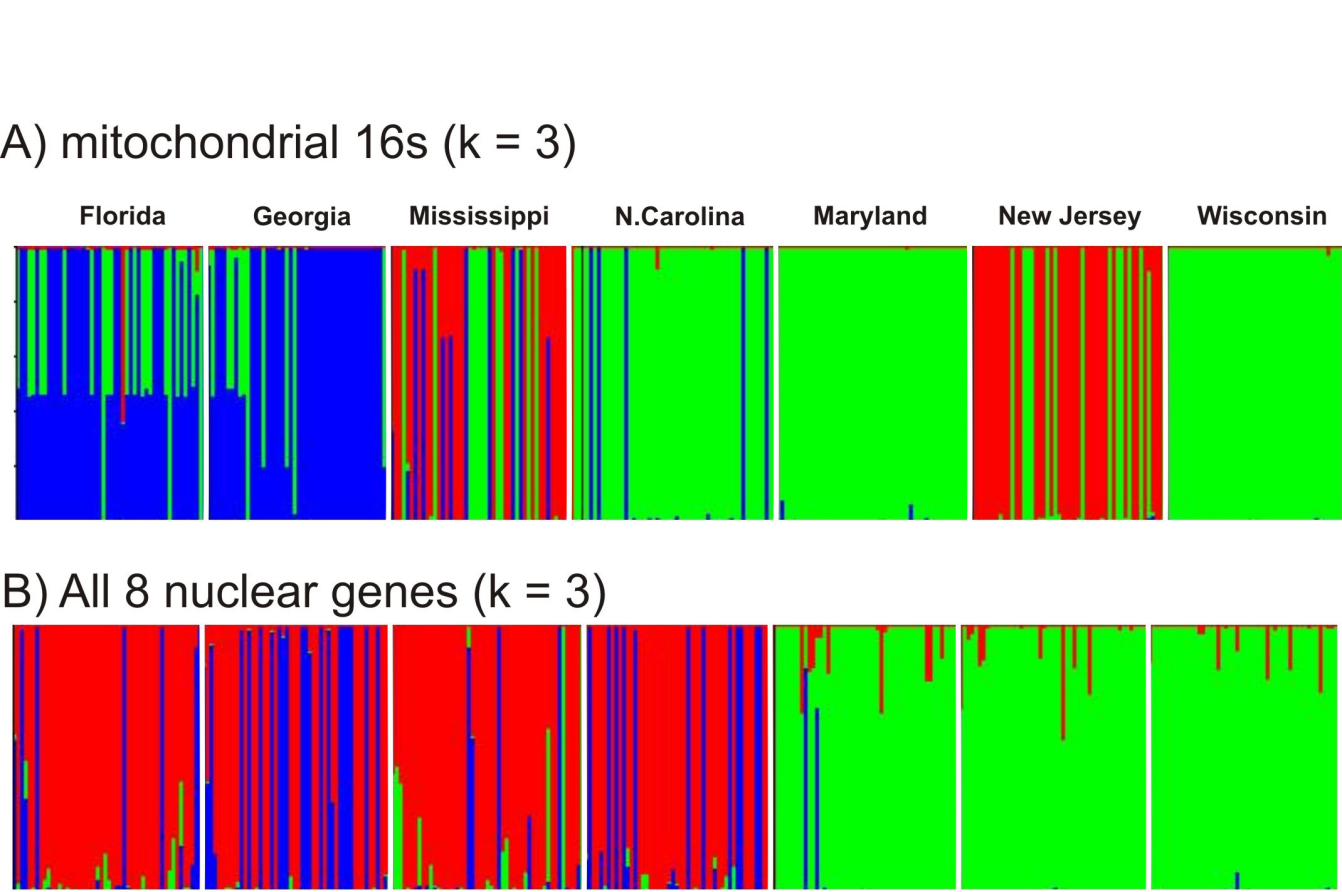
DISTRUCT plots created from STRUCTURE output. Cluster membership is represented as different colors, and individual ticks were depicted as vertical bars partitioned into segments that correspond to membership coefficients in each of the seven collection sites. The numbers of genetic clusters (K) of ticks was determined on the basis of their A) 16S haplotypes or B) for all 1,155 SNPs in the 8 nuclear genes combined.

The program STRUCTURE was used to estimate the numbers of genetic clusters (K) of ticks on the basis of their 16S haplotypes (Fig 7A) and for all 1,155 SNPs in the combined nuclear genes (Fig 7B). The ΔK method identified three genetic groups in the 16S dataset. Ticks in the first group (green bars) were mostly collected in Wisconsin, Maryland, and North Carolina. Ticks in the second group (red bars) were mostly ticks from New Jersey and Mississippi. Ticks in the third (blue) genetic group are mostly from Georgia and Florida. Ticks from the green group are mostly northern but do appear in the south while ticks from the blue group are mostly southern and do not appear in the north. On the other hand ticks from the red group occur exclusively in New Jersey and Mississippi. These results suggest historical unidirectional and bidirectional flow of mitochondrial genomes. Blue southern ticks appear in other southern states. Green ticks appear frequently in southern collections. Red ticks appear in both the northern and southern collections.

A different pattern emerges from analysis of the combined nuclear genes which reflects current gene flow. The ΔK method again estimated three genetic groups. Green bars represent ticks primarily from Maryland, New Jersey, and Wisconsin while red bars represent ticks primarily from the four southern collections. The third blue genetic group consists primarily of ticks from Georgia, with some blue group ticks appearing in Florida, Mississippi and North Carolina. Two blue group ticks appear in Maryland and some northern ticks have genes from the red group. Conversely northern green cluster genes appear variously in southern collections. These analyses suggest, in agreement with Fig 5, that there is limited current flow of nuclear genes between northern and southern collections.

### Nonequilibrium migration patterns among *I*. *scapularis* populations

Rates and directions of gene flow were estimated for all nuclear genes combined using LAMARC. Table 5 lists the average *4N*_*e*_ *m* among northern collections (6 values), among southern collections (12 values), from north to south (12 values) and south to north (12 values). When analyzing all nuclear genes together, the average *4N*_*e*_ *m* among northern collections was 6.42 reproductive migrants per generation while there were 5.27 reproductive migrants per generation among southern collections. *4N*_*e*_ *m* was reduced between southern and northern collections with 1.51 migrants from the north to the south/generation and only 0.07 migrants from the south into the north/generation. Considering all nuclear markers together there was ~ 21 fold greater migration from north to south relative to the reverse direction.

**Table 5:** Pairwise *4N*_*e*_ *m* and the lower 2.5% and upper 95% Highest Density Intervals (HDI) among all pairs of collections (42 pairs). Values at the base of the table are average *4N*_*e*_ *m* and their lower 2.5% and upper 95% HDI among northern collections (6 values), among southern collections (12 values), from north to south (12 values) and south to north (12 values).

From	Into	Direction	*4N*_*e*_ *m*	2.5%	97.5%
New Jersey	Maryland	Northern	0.000	0.000	0.000
New Jersey	Wisconsin	Northern	1.062	1.055	1.097
New Jersey	North Carolina	North to South	2.190	1.944	2.240
New Jersey	Georgia	North to South	7.390	6.938	7.480
New Jersey	Mississippi	North to South	0.000	0.000	0.000
New Jersey	Florida	North to South	0.000	0.000	0.000
Maryland	New Jersey	Northern	24.938	24.203	25.192
Maryland	Wisconsin	Northern	7.353	7.254	7.821
Maryland	North Carolina	North to South	0.904	0.903	0.904
Maryland	Georgia	North to South	0.000	0.000	0.000
Maryland	Mississippi	North to South	0.000	0.000	0.000
Maryland	Florida	North to South	4.806	4.781	5.011
Wisconsin	New Jersey	Northern	0.000	0.000	0.000
Wisconsin	Maryland	Northern	5.177	4.954	5.255
Wisconsin	North Carolina	North to South	0.000	0.000	0.000
Wisconsin	Georgia	North to South	0.000	0.000	0.000
Wisconsin	Mississippi	North to South	2.801	2.800	2.804
Wisconsin	Florida	North to South	0.000	0.000	0.000
North Carolina	New Jersey	South to North	0.000	0.000	0.000
North Carolina	Maryland	South to North	0.000	0.000	0.000
North Carolina	Wisconsin	South to North	0.000	0.000	0.000
North Carolina	Georgia	Southern	11.835	11.099	11.968
North Carolina	Mississippi	Southern	29.483	29.437	29.865
North Carolina	Florida	Southern	5.609	5.582	5.698
Georgia	New Jersey	South to North	0.000	0.000	0.000
Georgia	Maryland	South to North	0.000	0.000	0.000
Georgia	Wisconsin	South to North	0.000	0.000	0.000
Georgia	North Carolina	Southern	0.000	0.000	0.000
Georgia	Mississippi	Southern	8.561	8.468	8.954
Georgia	Florida	Southern	0.000	0.000	0.000
Mississippi	New Jersey	South to North	0.000	0.000	0.000
Mississippi	Maryland	South to North	0.313	0.312	0.314
Mississippi	Wisconsin	South to North	0.531	0.528	0.548
Mississippi	North Carolina	Southern	1.930	1.844	2.376
Mississippi	Georgia	Southern	2.957	2.775	2.992
Mississippi	Florida	Southern	0.000	0.000	0.000
Florida	New Jersey	South to North	0.000	0.000	0.000
Florida	Maryland	South to North	0.000	0.000	0.000
Florida	Wisconsin	South to North	0.000	0.000	0.000
Florida	North Carolina	Southern	0.000	0.000	0.000
Florida	Georgia	Southern	1.481	1.387	1.496
Florida	Mississippi	Southern	1.440	1.411	1.689
North	North	Averages	6.42	6.24	6.56
South	South	Averages	5.27	5.17	5.42
North	South	Averages	1.51	1.45	1.54
South	North	Averages	0.07	0.07	0.07

*4N*_*e*_ *m* values are also depicted as arrows in Fig 1 to indicate the direction and relative rate of gene flow between collection pairs. *4N*_*e*_ *m* <1 between pairs of collections are not connected with arrows. Green arrows among New Jersey, Maryland and Wisconsin indicate both directional and bidirectional gene flow among northern populations. Similarly, green arrows among North Carolina, Georgia, Mississippi and Florida indicate both directional and bidirectional gene flow among southern populations. There are no arrows that originate in the south and end in northern collections. Instead southern collections are all recipients of alleles from the north. North Carolina and Georgia receive immigrants from New Jersey, Florida receives immigrants from Maryland and Mississippi receives immigrants from Wisconsin.

## Discussion

Earlier studies on the population genetics of *I*. *scapularis* consistently reported greater genetic diversity in southern versus northern collections. This trend was noted earlier for the 16S mitochondrial genes [14–16] and including the 12S [14], and CO1 [25] mitochondrial genes. The same trend was also noted for three nuclear genes (serpin2, ixoderin B and lysozyme) [25] and for the same nuclear markers used in the present study [17]. The same trend was found in the current study and, along with Figs 4 and 6, recapitulates the common observation of greater genetic diversity in the regions of ancestral origin of a species.

Table 3 shows that only a small percentage of alleles are shared between northern and southern collections. This trend is also seen with the maximum clade credibility coalescent tree (Fig 5) and in Fig 7 wherein northern genetic group genes appeared in the south but few southern genetic group genes appear in the north. This same trend with nuclear genes was reported earlier [17]. The AMOVA results are similar in this and earlier studies in which genetic differences in nuclear genes between north and south accounted for 15.5% in the present study and accounted for 20% [25] and 18% [17] in earlier studies. Genetic differences in nuclear genes among northern and among southern collections accounted for 6% of the variation in in the current study and for 15% [25] and 6% [17] in previous studies. In all three studies, the differences between north and south were greater than the differences among northern or among southern collections. However the variance patterns in with the 16S differ from patterns in earlier studies which found 33.6%[15] to 16.8%[25] of variance arose between north and south while 27% [15] to 10.6% [25] of variance arose within northern and southern collections. This may reflect differences in sampling location in some states. For example Norris et al [14] sampled from Currituck Stokes county in coastal North Carolina(NC), Qiu et al [15] sampled from Currituck and Washington County in eastern NC, Sakamoto et al [25] sampled from Stokes county in western NC[25] and the current study sampled from Gates Co. (Fig 1) near both Currituck and Washington County in eastern NC. Ecological Units vary enormously from east to west in NC; extending from “Tidewater” in eastern NC to “Blue Ridge” in western NC [45, 46]. North Carolina ticks appeared more similar to northern genetic groups in the STRUCTURE analysis of the 16S (Fig 7).

Mismatch patterns were previously examined in northern and southern *I*. *scapularis* collections [15, 25]. These studies also found a unimodal distribution with a maximum of 4 mismatches in northern *I*. *scapularis* collections and detected a multimodal distribution with up to 17 mismatches in southern collections [15]. The mismatch patterns among CO1 genes in southern collections were multimodal with mismatches as high as 61 but were unimodal with up to 15 mismatches in northern collections [25]. Thus southern *I*. *scapularis* populations appear to be at a demographic equilibrium while northern populations have gone through a recent demographic expansion. This trend is in agreement with the Tajima’s D analysis in the current and previous [25] studies which also suggested recent population expansion. This conclusion is also supported by the EBSP analyses wherein southern tick populations have been expanding over the last 500,000 years while the northern populations only began expanding within the last 20,000 to 50,000 years.

The maximum clade credibility coalescent 16S tree implies historical unidirectional introgression of southern ticks into the north and agrees in almost all respects with the published trees estimated by Maximum Parsimony, Distance/Neighbor Joining, Maximum Likelihood and Bayesian techniques in all previous studies [12–17, 25]. In contrast, the maximum clade credibility coalescent tree for the combined nuclear genes implies very little current gene flow between northern and southern ticks.

Taken as a whole these results suggest a scenario in which *I*. *scapularis* arose as a species in the southeastern U.S. Populations in Mississippi and Georgia began expanding into Florida and North Carolina half a million years ago and into Maryland and New Jersey only 50,000 years ago and with an accompanying population bottleneck. Wisconsin populations only began expanding in the last 20,000 years as the Laurentide Ice Sheet retreated. During the northward expansion and accompanying population bottleneck, a barrier to gene flow arose such that very little bidirectional gene flow occurred between northern and southern ticks. This barrier must have existed over an extended period of time (and may still exist) in order to account for the small percentage of the alleles shared between northern and southern collections and for the patterns of isolation implied by the nuclear maximum clade credibility coalescent tree. During this time *I*. *scapularis* nymphs may have evolved away from an ancestral tendency to quest at ground level towards questing above the leaf litter. At the same time they may have switched from the ancestral tendency to seek and feed on reptiles towards locating and feeding primarily on mammals. Eventually the gene flow barrier seems to have partially broken down such that there is now gene flow albeit unidirectional from northern populations into the south.

This scenario assumes that questing above the leaf litter and host preference are both genetic traits which are polymorphic in natural populations. In fact there is no data to support this idea. These behaviors may instead be plastic in response to different environmental conditions in northern and southern habitats.

While our study supports a model of current restricted, largely unidirectional gene flow, many biological questions remain unanswered. The principal vectors of Lyme disease spirochetes in the eastern US are nymphal *I*. *scapularis*. By questing above the leaf litter, they frequently contact humans and rodents, thereby serving as an excellent bridge vectors passing Lyme disease spirochetes from their rodent and soricid reservoirs to people [47]. In contrast, southern nymphs quest mainly below the leaf litter and feed mainly on reptiles [48, 49], which are considered sink-hosts, therefore interrupting the transmission of spirochetes. Could this behavior impact rates of migration and gene flow between populations in certain areas?

Apparently, the highly successful populations of *I*. *scapularis* in the northeastern United States are favored by temperate environmental conditions and abundant rodent, bird, and large mammal hosts. Earlier studies [20, 21] and the current study demonstrate that these ticks are spreading to the Midwestern United States and the southern United States. They are also spreading northward to Canada [50–53], perhaps on birds. The spread of northern vectorial competent ticks to the southern United States would be of public health concern, if questing and host finding patterns have a strong genetic component, as opposed to these behaviors being merely temporal adaptations to extant local environmental conditions. More intensive geographic sampling of *I*. *scapularis* especially in Virginia, the Carolinas and Georgia will be needed to delineate the geographic regions where barriers and corridors for gene flow are occurring.

## Supporting Information

S1 Table Membership coefficients (inferred ancestry) for individual ticks as estimated in STRUCTURE [35]. Colors correspond to those in Fig 7B. In individual labels FL = Florida, GA = Georgia, MD = Maryland, MS = Mississippi, NC = North Carolina, NJ = New Jersey, and WI = Wisconsin.
(XLSX)

## References

[1] Kuehn BM. CDC Estimates 300 000 US Cases of Lyme Disease Annually. Jama-J Am Med Assoc. 2013;310(11):1110–. . 10.1001/jama.2013.278331 24045727

[2] Schauber EM, Gertz SJ, Maple WT, Ostfeld RS. Coinfection of blacklegged ticks (Acari: Ixodidae) in Dutchess County, New York, with the agents of Lyme disease and human granulocytic ehrlichiosis. J Med Entomol. 1998;35(5):901–3. . 9775627 10.1093/jmedent/35.5.901

[3] Sr Telford, Armstrong P, Katavolos P, Foppa I, Garcia A, Wilson M, et al A new tick-borne encephalitis-like virus infecting New England deer ticks, Ixodes dammini. Emerg Infect Dis. 1997;3(2):165–70. . 9204297 10.3201/eid0302.970209 PMC2627606

[4] Spielman A, Wilson ML, Levine JF, Piesman J. Ecology of Ixodes dammini-borne human babesiosis and Lyme disease. Annu Rev Entomol. 1985;30:439–60. Epub 1985/01/01. 10.1146/annurev.en.30.010185.002255 . 3882050

[5] Dennis DT, Nekomoto TS, Victor JC, Paul WS, Piesman J. Reported distribution of Ixodes scapularis and in Ixodes pacificus (Acari: Ixodidae) in the United States. J Med Entomol. 1998;35(5):629–38. . 9775584 10.1093/jmedent/35.5.629

[6] Orloski K, Hayes E, Campbell G, Dennis D. Surveillance for Lyme disease—United States, 1992–1998. MMWR CDC Surveill Summ. 2000;49(3):1–11. . 10817483

[7] Dennis DT. Rash decisions: Lyme disease, or not? Clin Infect Dis. 2005;41(7):966–8. Epub 2005/09/06. 10.1086/432958 . 16142660

[8] Diuk-Wasser MA, Vourc'h G, Cislo P, Hoen AG, Melton F, Hamer SA, et al Field and climate-based model for predicting the density of host-seeking nymphal Ixodes scapularis, an important vector of tick-borne disease agents in the eastern United States. Global Ecol Biogeogr. 2010;19(4):504–14. 10.1111/j.1466-8238.2010.00526.x .

[9] Diuk-Wasser MA, Hoen AG, Cislo P, Brinkerhoff R, Hamer SA, Rowland M, et al Human Risk of Infection with Borrelia burgdorferi, the Lyme Disease Agent, in Eastern United States. Am J Trop Med Hyg. 2012;86(2):320–7. 10.4269/ajtmh.2012.11-0395 . 22302869 PMC3269287

[10] Pepin KM, Eisen RJ, Mead PS, Piesman J, Fish D, Hoen AG, et al Geographic Variation in the Relationship between Human Lyme Disease Incidence and Density of Infected Host-Seeking Ixodes scapularis Nymphs in the Eastern United States. Am J Trop Med Hyg. 2012;86(6):1062–71. 10.4269/ajtmh.2012.11-0630 . 22665620 PMC3366524

[11] Oliver JH, Owsley MR, Hutcheson HJ, James AM, Chen CS, Irby WS, et al Conspecificity of the Ticks Ixodes-Scapularis and Ixodes-Dammini (Acari, Ixodidae). J Med Entomol. 1993;30(1):54–63. . 8433346 10.1093/jmedent/30.1.54

[12] Wesson DM, Mclain DK, Oliver JH, Piesman J, Collins FH. Investigation of the Validity of Species Status of Ixodes-Dammini (Acari, Ixodidae) Using Rdna. P Natl Acad Sci USA. 1993;90(21):10221–5. 10.1073/pnas.90.21.10221 . PMC47746 8234280

[13] Caporale DA, Rich SM, Spielman A, Telford SR, Kocher TD. Discriminating between Ixodes ticks by means of mitochondrial DNA sequences. Mol Phylogenet Evol. 1995;4(4):361–5. 10.1006/mpev.1995.1033 . 8747292

[14] Norris DE, Klompen JSH, Keirans JE, Black WC. Population genetics of Ixodes scapularis (Acari: Ixodidae) based on mitochondrial 16S and 12S genes. J Med Entomol. 1996;33(1):78–89. . 8906909 10.1093/jmedent/33.1.78

[15] Qiu WG, Dykhuizen DE, Acosta MS, Luft BJ. Geographic uniformity of the Lyme disease spirochete (Borrelia burgdorferi) and its shared history with tick vector (Ixodes scapularis) in the northeastern United States. Genetics. 2002;160(3):833–49. . 11901105 10.1093/genetics/160.3.833 PMC1462027

[16] Rich SM, Caporale DA, Telford SR, Kocher TD, Hartl DL, Spielman A. Distribution of the Ixodes-Ricinus-Like Ticks of Eastern North-America. P Natl Acad Sci USA. 1995;92(14):6284–8. 10.1073/pnas.92.14.6284 . PMC41502 7603983

[17] Van Zee J, Black WC, Levin M, Goddard J, Smith J, Piesman J. High SNP density in the blacklegged tick, Ixodes scapularis, the principal vector of Lyme disease spirochetes. Ticks Tick-Borne Dis. 2013;4(1–2):63–71. 10.1016/j.ttbdis.2012.07.005 . 23219364

[18] Brinkerhoff RJ, Gilliam WF, Gaines D. Lyme disease, Virginia, USA, 2000–2011. Emerg Infect Dis. 2014;20(10):1661–8. 10.3201/eid2010.130782 25272308 PMC4193267

[19] Khatchikian CE, Prusinski M, Stone M, Backenson PB, Wang IN, Levy MZ, et al Geographical and environmental factors driving the increase in the Lyme disease vector Ixodes scapularis. Ecosphere. 2012;3(10). 10.1890/ES12-00134.1 24371541 PMC3872055

[20] Kelly RR, Gaines D, Gilliam WF, Brinkerhoff RJ. Population genetic structure of the Lyme disease vector Ixodes scapularis at an apparent spatial expansion front. Infect Genet Evol. 2014;27:543–50. 10.1016/j.meegid.2014.05.022 . 24882702

[21] Humphrey PT, Caporale DA, Brisson D. Uncoordinated phylogeography of Borrelia burgdorferi and its tick vector, Ixodes scapularis. Evolution. 2010;64(9):2653–63. 10.1111/j.1558-5646.2010.01001.x 20394659 PMC2919648

[22] Araya-Anchetta A, Busch JD, Scoles GA, Wagner DM. Thirty years of tick population genetics: A comprehensive review. Infection, Genetics and Evolution. 2015;29(0):164–79. doi: 10.1016/j.meegid.2014.11.008. 25461844

[23] McLain DK, Wesson DM, Collins FH, Oliver JH. Evolution of the rDNA spacer, ITS 2, in the ticks Ixodes scapularis and I. pacificus (Acari: Ixodidae). Heredity (Edinb). 1995;75 (Pt 3):303–19. . 7558890 10.1038/hdy.1995.139

[24] Qiu WG, Dykhuizen DE, Acosta MS, Luft BJ. Geographic uniformity of the Lyme disease spirochete (Borrelia burgdorferi) and its shared history with tick vector (Ixodes scapularis) in the Northeastern United States. Genetics. 2002;160(3):833–49. 11901105 10.1093/genetics/160.3.833 PMC1462027

[25] Sakamoto JM, Goddard J, Rasgon JL. Population and Demographic Structure of Ixodes scapularis Say in the Eastern United States. Plos One. 2014;9(7). doi: ARTN e101389 10.1371/journal.pone.0101389 PMC4099084 25025532

[26] Excoffier L, Slatkin M. Maximum-Likelihood-Estimation of Molecular Haplotype Frequencies in a Diploid Population. Mol Biol Evol. 1995;12(5):921–7. . 7476138 10.1093/oxfordjournals.molbev.a040269

[27] Drummond AJ, Rambaut A. BEAST: Bayesian evolutionary analysis by sampling trees. Bmc Evol Biol. 2007;7. doi: Artn 214 10.1186/1471-2148-7-214 . 17996036 PMC2247476

[28] Kuhner MK. Coalescent genealogy samplers: windows into population history. Trends Ecol Evol. 2009;24(2):86–93. 10.1016/j.tree.2008.09.007 . 19101058 PMC4714702

[29] Librado P, Rozas J. DnaSP v5: a software for comprehensive analysis of DNA polymorphism data. Bioinformatics. 2009;25(11):1451–2. 10.1093/bioinformatics/btp187 . 19346325

[30] Stephens M, Smith NJ, Donnelly P. A New Statistical Method for Haplotype Reconstruction from Population Data. American Journal of Human Genetics. 2001;68(4):978–89. . 11254454 10.1086/319501 PMC1275651

[31] Tajima F. Statistical-Method for Testing the Neutral Mutation Hypothesis by DNA Polymorphism. Genetics. 1989;123(3):585–95. . 2513255 10.1093/genetics/123.3.585 PMC1203831

[32] Stamatakis A. RAxML version 8: a tool for phylogenetic analysis and post-analysis of large phylogenies. Bioinformatics. 2014;30(9):1312–3. 10.1093/bioinformatics/btu033 . 24451623 PMC3998144

[33] Rogers AR, Harpending H. Population-Growth Makes Waves in the Distribution of Pairwise Genetic-Differences. Mol Biol Evol. 1992;9(3):552–69. . 1316531 10.1093/oxfordjournals.molbev.a040727

[34] Excoffier L, Laval G, Schneider S. Arlequin (version 3.0): An integrated software package for population genetics data analysis. Evol Bioinform. 2005;1:47–50. . PMC2658868 19325852

[35] Pritchard JK, Stephens M, Donnelly P. Inference of population structure using multilocus genotype data. Genetics. 2000;155(2):945–59. PubMed PMID: WOS:000087475100039. 10835412 10.1093/genetics/155.2.945 PMC1461096

[36] Gilbert KJ, Andrew RL, Bock DG, Franklin MT, Kane NC, Moore JS, et al Recommendations for utilizing and reporting population genetic analyses: the reproducibility of genetic clustering using the program STRUCTURE (vol 21, pg 4925, 2012). Molecular ecology. 2013;22(8):2357–. 10.1111/mec.12248 . 22998190

[37] Evanno G, Regnaut S, Goudet J. Detecting the number of clusters of individuals using the software STRUCTURE: a simulation study. Molecular ecology. 2005;14(8):2611–20. Epub 2005/06/23. 10.1111/j.1365-294X.2005.02553.x . 15969739

[38] Rosenberg NA. DISTRUCT: a program for the graphical display of population structure. Mol Ecol Notes. 2004;4(1):137–8. 10.1046/j.1471-8286.2003.00566.x .

[39] Ronquist F, Teslenko M, van der Mark P, Ayres DL, Darling A, Hohna S, et al MrBayes 3.2: Efficient Bayesian Phylogenetic Inference and Model Choice Across a Large Model Space. Syst Biol. 2012;61(3):539–42. 10.1093/sysbio/sys029 . 22357727 PMC3329765

[40] Kingman JFC. Origins of the coalescent: 1974–1982. Genetics. 2000;156(4):1461–3. . 11102348 10.1093/genetics/156.4.1461 PMC1461350

[41] Hasegawa M, Kishino H, Yano TA. Dating of the Human Ape Splitting by a Molecular Clock of Mitochondrial-DNA. J Mol Evol. 1985;22(2):160–74. 10.1007/Bf02101694 . 3934395

[42] Heled J, Drummond AJ. Bayesian inference of population size history from multiple loci. Bmc Evol Biol. 2008;8. doi: Artn 289 10.1186/1471-2148-8-289 . PMC2636790 18947398

[43] Kuhner MK. LAMARC 2.0: maximum likelihood and Bayesian estimation of population parameters. Bioinformatics. 2006;22(6):768–70. 10.1093/bioinformatics/btk051 . 16410317

[44] Papadopoulou A, Anastasiou I, Vogler AP. Revisiting the Insect Mitochondrial Molecular Clock: The Mid-Aegean Trench Calibration. Mol Biol Evol. 2010;27(7):1659–72. 10.1093/molbev/msq051 . 20167609

[45] United States. Forest Service., Keys JE, United States. Forest Service. Southern Region. Geometronics. Ecological units of the eastern United States first approximation. Atlanta, Ga.: The Service,; 1995. p. 1 computer laser optical disc col. maps 4 3/4 in.

[46] United States. Forest Service. Southern Region. Geometronics., Keys JE, Carpenter CA, cartographers. Ecological units of the eastern United States: first approximation [1 map]. Atlanta, Ga.?: U.S. Dept. of Agriculture, Forest Service,; 1995.

[47] Piesman J. Ecology of Borrelia burgdorferi sensu lato in North America In: Gray JS, Kahl O, Lane RS, Stanek G, editors. Lyme Borreliosis: Biology, Epidemiology, and Control Wallingford, Oxon.: CABI Publishing,; 2002.

[48] Gatewood AG, Liebman KA, Vourc'h G, Bunikis J, Hamer SA, Cortinas R, et al Climate and Tick Seasonality Are Predictors of Borrelia burgdorferi Genotype Distribution. Appl Environ Microb. 2009;75(8):2476–83. 10.1128/Aem.02633-08 . PMC2675205 19251900

[49] Oliver JH. Lyme borreliosis in the southern United States: A review. J Parasitol. 1996;82(6):926–35. 10.2307/3284201 . 8973401

[50] Koffi JK, Leighton PA, Pelcat Y, Trudel L, Lindsay LR, Milord F, et al Passive Surveillance for I. scapularis Ticks: Enhanced Analysis for Early Detection of Emerging Lyme Disease Risk. J Med Entomol. 2012;49(2):400–9. 10.1603/Me11210 . 22493860

[51] Ogden NH, Lindsay RL, Hanincova K, Barker IK, Bigras-Poulin M, Charron DF, et al Role of migratory birds in introduction and range expansion of Ixodes scapularis ticks and of Borrelia burgdorferi and Anaplasma phagocytophilum in Canada (vol 74, pg 1780, 2008). Appl Environ Microb. 2008;74(12):3919–. 10.1128/Aem.00857-08 . PMC2268299 18245258

[52] Leighton PA, Koffi JK, Pelcat Y, Lindsay LR, Ogden NH. Predicting the speed of tick invasion: an empirical model of range expansion for the Lyme disease vector Ixodes scapularis in Canada. J Appl Ecol. 2012;49(2):457–64. 10.1111/j.1365-2664.2012.02112.x .

[53] Ogden NH, Maarouf A, Barker IK, Bigras-Poulin M, Lindsay LR, Morshed MG, et al Climate change and the potential for range expansion of the Lyme disease vector Ixodes scapularis in Canada. Int J Parasitol. 2006;36(1):63–70. 10.1016/j.ijpara.2005.08.016 . 16229849

